# Clinical, radiological, therapeutic and prognostic differences between MOG-seropositive and MOG-seronegative pediatric acute disseminated encephalomyelitis patients: a retrospective cohort study

**DOI:** 10.3389/fnins.2023.1128422

**Published:** 2023-05-19

**Authors:** Xueshan Dong, Yan Jiang, Ping Yuan, Xiao Fan, Jiannan Ma, Peng Wu, Li Jiang, Xiujuan Li

**Affiliations:** ^1^Department of Neurology, Children's Hospital of Chongqing Medical University, National Clinical Research Center for Child Health and Disorders, Ministry of Education Key Laboratory of Child Development and Disorders, Chongqing, China; ^2^China International Science and Technology Cooperation Base of Child Development and Critical Disorders, Chongqing, China; ^3^Chongqing Key Laboratory of Pediatrics, Chongqing, China; ^4^Department of Radiology, Children's Hospital of Chongqing Medical University, National Clinical Research Center for Child Health and Disorders, Ministry of Education Key Laboratory of Child Development and Disorders, Chongqing, China

**Keywords:** acute disseminated encephalomyelitis, ADEM, myelin oligodendrocyte glycoprotein, MOG, prognosis, pediatric

## Abstract

**Objective:**

This study aimed to compare the clinical, radiological, therapeutic, and prognostic differences between pediatric patients showing acute disseminated encephalomyelitis (ADEM) with and without myelin oligodendrocyte glycoprotein (MOG) antibodies.

**Methods:**

We retrospectively collected all available data of children diagnosed with ADEM and tested for serum MOG antibodies at the Children's Hospital of Chongqing Medical University from January 2017 to May 2021.

**Results:**

A total of 62 patients were included in our cohort, of which 35 were MOG-seropositive and 27 were MOG-seronegative. MOG-seropositive ADEM children presented with significantly lower rates of seizures (*P* = 0.038) and cranial nerve (III–XII) palsy (*P* = 0.003). Isolated leukocytosis in the blood was more common in ADEM children with MOG antibodies (*P* < 0.001). The two groups showed no significant differences in the distributions and extent of the MRI lesions as well as the appearance of typical/atypical magnetic resonance imaging (MRI) features. MOG-seropositive children were more likely to relapse (*P* = 0.017) despite having slower oral prednisolone tapering after acute treatments (*P* = 0.028). In scoring performed on the basis of two neurological function scoring systems, MOG-seropositive children showed milder neurological disability status at onset (*P* = 0.017 and 0.025, respectively) but showed no difference during follow-up.

**Conclusion:**

In summary, the differences in the clinical manifestations and auxiliary examination findings for MOG-seropositive and MOG-seronegative ADEM children lacked significance and specificity, making early identification difficult. MOG-seropositive children were more likely to relapse and showed slower steroid tapering. Moreover, MOG-seronegative children tended to have more severe neurological impairments at onset with no difference during follow-up.

## 1. Introduction

Acute disseminated encephalomyelitis (ADEM), a common form of acquired demyelinating syndrome (ADS), is a rare, first-episode inflammatory demyelinating disease of the central nervous system (CNS), that is dominant in children (Menge et al., 2005; Krupp et al., 2013). Most children with ADEM have a transient monophasic course with a favorable prognosis, but recurrence including multiphasic disseminated encephalomyelitis and ADEM followed by recurrent optic neuritis, and even death still occur (Leake et al., 2004; Mikaeloff et al., 2007; Cole et al., 2019). The lack of sensitive and specific magnetic resonance imaging (MRI) criteria, as well as serum/cerebrospinal fluid (CSF) biomarkers, has made it difficult to distinguish ADEM from other acquired demyelinating syndromes and predict the prognosis (Brenton and Banwell, 2016).

Myelin oligodendrocyte glycoprotein antibodies (MOG-abs) have been recently detected in patients with various types of acquired demyelinating syndromes, including ADEM (Hennes et al., 2017), and have become a hot topic of research. MOG-abs can be detected in more than 50% of children with ADEM, while they are present in almost all children with multiphasic disseminated encephalomyelitis (Reindl and Waters, 2019; Bruijstens et al., 2020). One study on MOG-ab-associated disease (MOGAD) showed that 38% of children with MOGAD showing persistently positive serum MOG-abs relapsed, while only 13% of MOGAD children whose serum MOG-abs turned negative had recurrence, suggesting that MOG-abs could be significantly associated with the recurrence and prognosis of acquired demyelinating syndromes (Waters et al., 2020).

The association between MOG-abs and ADEM as well as its related recurrence forms has been gradually recognized (Wong et al., 2018). However, the pathophysiological role of MOG-abs in ADEM remains under investigation and the association with clinical characteristics and prognosis is still unclear (Otallah, 2020). Currently, most clinical and fundamental studies that focus on the MOG-seropositive population have described and discussed the characteristics of MOG-ab-associated ADEM, with limited attention to the MOG-seronegative patients. As a result, differences in the clinical manifestations, imaging characteristics, recurrence, and prognosis between children with MOG-seropositive and MOG-seronegative ADEM remain unclear.

In this study, to clinically explore the role of MOG-abs in the diagnosis and treatment of ADEM and evaluate its value in guiding treatments and predicting prognosis, we retrospectively compared the differences in demographic characteristics, clinical characteristics, imaging findings, treatments, and prognosis between MOG-seropositive and MOG-seronegative pediatric patients with ADEM.

## 2. Materials and methods

### 2.1. Patients

**Figure 1:**
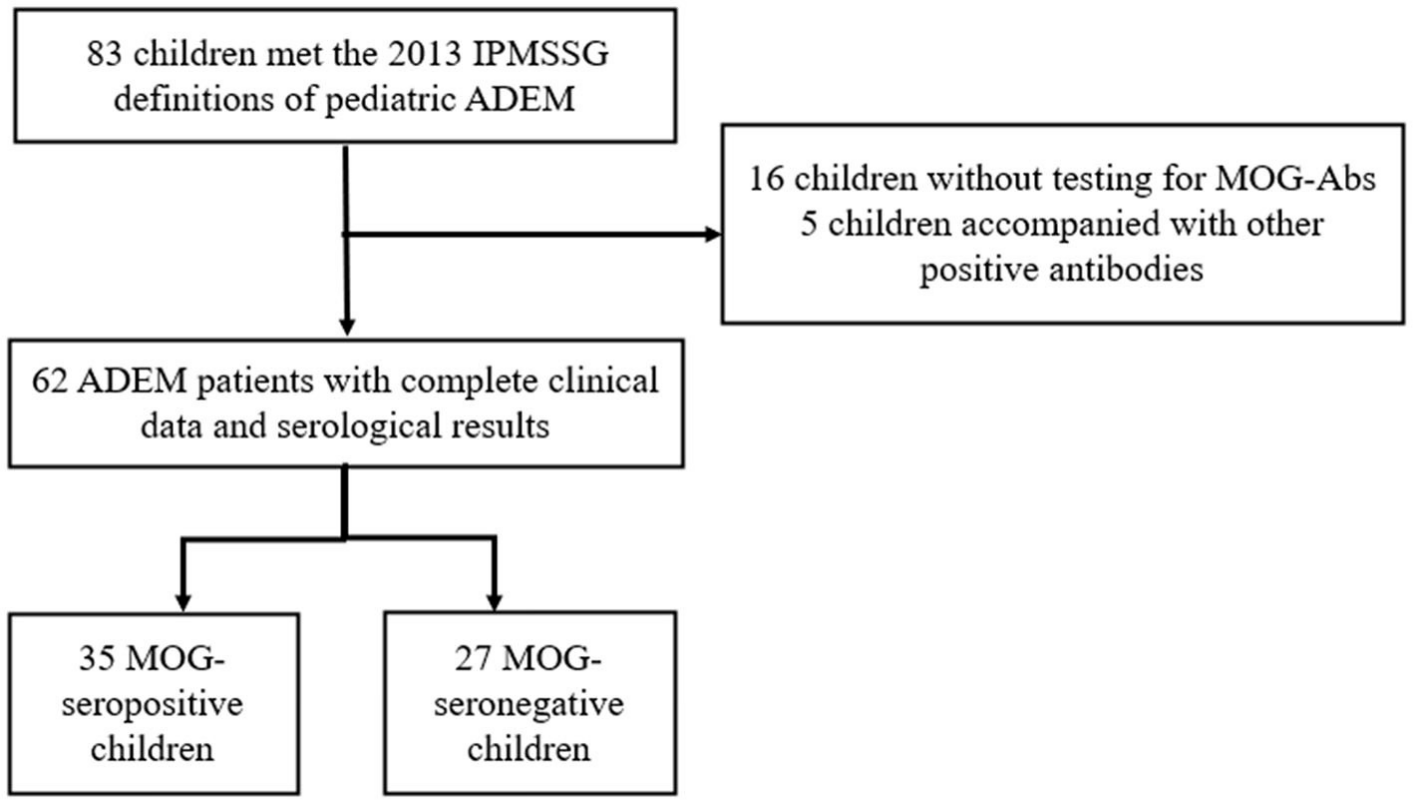
Flowchart of patient inclusion and exclusion.

Our study was approved by the Ethics Committee of the Children's Hospital of Chongqing Medical University. Written informed consent to participate in this study was provided by the participants' legal guardians or next of kin. We retrospectively screened all the children hospitalized in the Children's Hospital of Chongqing Medical University from January 2017 to May 2021. Children presenting with their first CNS demyelination event and diagnosed as ADEM according to the 2013 International Pediatric Multiple Sclerosis Study Group (IPMSSG) criteria (Krupp et al., 2013) were included in our study. Patients who did not undergo testing for serum MOG-abs or had incomplete clinical data were excluded from our study. Moreover, in order to focus on the differences caused by MOG-abs, patients showing positive results for other specific antibodies such as those against aquaporin 4 (AQP4), N-methyl-D-aspartate receptor (NMDAR), or glial fibrillary acidic protein (GFAP) were also excluded from this study. The flowchart for patient screening and inclusion is shown in Figure 1.

We collected general data, including information regarding the patient's name, sex, ID number, and age of onset; clinical manifestations such as prodromal factors and clinical symptoms and signs; data for the titers of MOG-abs in serum/CSF and the results of other laboratory examinations; and imaging information such as MRI and electroencephalogram (EEG) findings and information regarding acute-phase treatments during hospitalization. Meanwhile, changes in the MOG antibody (MOG-ab) status and prognosis of patients, including recovery and recurrence, were followed up after discharge.

### 2.2. Detection of serum MOG antibody status

All serum specimens in our study were evaluated by cell-based assays (CBAs), which are officially and widely recommended in clinical settings (Jarius et al., 2018). The method involves transfecting the co-expressed human full-length MOG plasmid into human embryonic kidney-293T (HEK293T) cells for 36 h (with a dilution ratio of 1:10), incubating the patient's serum and fluorescent secondary antibody in turn, and detecting the MOG antibody in the patient's serum by indirect immunofluorescence method. A total of two independent assessors evaluated each sample on the basis of the intensity of surface immunofluorescence. Titer levels of ≥1:10 were considered to indicate seropositivity.

### 2.3. MRI analysis

All MRI images evaluated in this study were obtained with a 1.5T or 3.0T MRI unit, including the following sequences: T1-weighted, T2-weighted, fluid-attenuated inversion recovery (FLAIR), and T1-weighted post-contrast (gadolinium) sequences. All included patients underwent cranial MRI examinations within 1 month after symptom onset and met the MRI diagnostic criteria for ADEM, and some patients also underwent spinal cord MRI. A total of 56 children in our study underwent cranial MRI at our hospital and had complete detailed imaging data available. MRI results were interpreted by two pediatric radiologists who independently read the images and came to conclusions without knowledge of the clinical conditions. In 54 of the 56 children, we performed follow-up MRI examinations for at least 3 months, and the two independent radiologists then assessed the MRI recovery status against the initial images.

We divided the distributions of MRI lesions into five regions, namely, the supratentorial white matter, deep gray matter, brainstem, cerebellum, and spinal cord. On the basis of this distribution, the extent of involvement was rated from 1 to 5, indicating the widespread score. Typical/atypical MRI lesion features have been defined in previous reports (Mikaeloff et al., 2004; Krupp et al., 2013).

### 2.4. Prognostic evaluations

Prognostic assessments in our study generally included evaluations of clinical recovery, MRI recovery, neurological dysfunction status, neurological sequelae, recurrence, and the use of maintenance treatments.

Clinical recovery was categorized as complete recovery (no neurological symptoms), mild residuals (mild residual symptoms, such as bladder dysfunction, excessive fatigue, and mild gross motor symptoms/signs, but much improved), or moderate/severe residuals (persistent significant neurological symptoms or sequelae such as motor dysfunction, secondary epilepsy, or cognitive impairment or mental disorders), and death. MRI recovery was also graded as complete resolution (no marked lesions remained), minor residuals (few remaining T2 signal lesions but much improved), moderate residuals (only slight improvement of T2 signal changes), and marked residuals (for example, atrophy) (Baumann et al., 2015; Cole et al., 2019).

We also used Expanded Disability Status Scale (EDSS) scores and modified Rankin Scale (mRS) scores to evaluate the disability status, especially the neurological dysfunction status of these patients at onset and during follow-up. Recurrence is defined as the new appearance or re-emergence of prior clinical symptoms and imaging lesions 3 months after onset.

### 2.5. Statistical analysis

Statistical analysis was performed using IBM SPSS, release v.23.0 (IBM Corporation). Comparison of continuous variables between groups was performed by Student's *t*-test or the Mann–Whitney *U*-test. Categorical variables were compared by the chi-square (χ^2^) test, Fisher's exact test, or sometimes the Mann–Whitney *U*-test. Statistical significance was defined as a two-sided *p*-value of < 0.05.

## 3. Results

### 3.1. Patients and general features

A total of 62 children diagnosed with ADEM (32 boys and 30 girls) were enrolled in our cohort after excluding 16 patients who were not tested for MOG-abs and five patients simultaneously showing positive results for other GFAP or NMDAR antibodies (Figure 1). In total, 35 (56.5%) of the 62 patients tested for MOG-abs were recorded as seropositive. The children in our study had a median age of 6 years [interquartile range (IQR), 4–9 years]. The demographic and clinical characteristics and findings of laboratory and neuroelectrophysiological examinations of these patients are shown in Table 1. All the patients were divided into two groups by MOG-ab status, with one group including 35 MOG-seropositive children and the other containing 27 MOG-seronegative children. The age and sex distributions of these two groups were similar. The MOG-seropositive group included 20 girls and 15 boys with a median age of 6 years (IQR, 4–10 years), and the MOG-seronegative group included 10 girls and 17 boys with a median age of 7 years (IQR, 4–9 years).

**Table 1:** Demographic and clinical features, laboratory tests, and neuroelectrophysiological examinations in ADEM children with and without MOG-abs.

	**MOG-ab- positive (*n* = 35)**	**MOG-ab-negative (*n* = 27)**	**Z/χ^2^**	***P***
Females, *n* (%)	20 (57.1)	10 (37.0)	2.467	0.116^#^
Age of onset (y), median (IQR)	6 (4–10)	7 (4–9)	−0.407	0.684^*^
Length of hospital stay (d), median (IQR)	17 (12–22)	16 (13–22)	−0.242	0.809^*^
**Prior events**, ***n*** **(%)**	**18 (51.4)**	**18 (66.7)**	**1.453**	**0.228** ^#^
Prodromal infection	15 (42.9)	17 (63.0)	2.467	0.116^#^
Vaccination	3 (8.6)	3 (11.1)	–	1.000^§^
**Symptoms at onset**, ***n*** **(%)**				
Fever	26 (74.3)	15 (55.6)	2.387	0.122^#^
Altered consciousness	33 (94.3)	26 (96.3)	–	1.000^§^
Emotional/behavioral changes	10 (28.6)	8 (29.6)	0.008	0.927^#^
Decreased muscle strength	24 (68.6)	21 (77.8)	0.649	0.420^#^
Headache	18 (51.4)	13 (48.1)	0.066	0.798^#^
Seizures	5 (14.3)	10 (37.0)	4.302	0.038^#^
CN (III–XII) palsy	9 (25.7)	17 (63.0)	8.685	0.003^#^
Ataxia	17 (48.6)	10 (37.0)	0.825	0.364^#^
Optic neuritis	4 (11.4)	5 (18.5)	0.178	0.673^#^
Sensory symptoms	11 (31.4)	9 (33.3)	0.025	0.874^#^
Bladder/rectum dysfunction	9 (25.7)	11 (40.7)	1.575	0.209^#^
**Blood tests**, ***n*** **(%)**				
WBC elevation	25 (74.1)	6 (22.2)	14.762	< 0.001^#^
CRP elevation	7 (20.0)	3 (11.1)	0.354	0.552^#^
PCT elevation	12/19 (63.2)	9/14 (64.3)	–	1.000^§^
Ferritin elevation	6/19 (31.6)	0/12 (0)	–	0.059^§^
Abnormal autoantibodies	7/26 (26.9)	7/17 (41.2)	0.951	0.329^#^
Abnormal immune indicators	6/15 (40.0)	8/13 (61.5)	–	0.449^§^
Thyroid dysfunction	5/15 (33.3)	4/8 (50.0)	–	0.657^§^
**CSF tests**, ***n*** **(%)**				
WBC elevation	20 (57.1)	15 (55.6)	0.016	0.901^#^
Elevated WBC level (^*^10^6^/L), median (IQR)	86 (31–123)	38 (23–104)	−1.219	0.227^*^
Lymphocytosis	13/19 (68.4)	12/15 (80.0)	–	0.697^§^
Protein elevation	13 (37.1)	12 (44.4)	0.338	0.561^#^
Elevated protein level (g/L), median (IQR)	0.60 (0.50–0.70)	0.65 (0.53–1.03)	−1.225	0.225^*^
OCBs	1/18 (5.6)	2/14 (14.3)	–	0.568^§^
**EEG abnormalities, *n* (%)**	**18/28 (64.3)**	**20/23 (87.0)**	**3.417**	**0.065^#^**
Slow background activity	18/28 (64.3)	20/23 (87.0)	3.417	0.065^#^
Interictal epileptic paroxysms	3/28 (10.7)	4/23 (17.4)	0.079	0.779^#^
**Abnormal VEP, *n* (%)**	**11/18 (61.1)**	**6/14 (42.9)**	**–**	**0.476^§^**

ADEM, acute disseminating encephalomyelitis; MOG-abs, myelin oligodendrocytes glycoprotein antibodies; IQR, interquartile range; CN, cranial nerves; CSF, cerebrospinal fluid; OCBs, oligoclonal bands; EEG, electroencephalogram; VEP, visual evoked potential.

^#^Using chi-square test.

^*^Using Mann–Whitney U-test.

^§^Using Fisher's exact test.

### 3.2. Clinical characteristics and auxiliary examinations

Approximately half of the children in both groups had at least one kind of prior event, including prodromal infections and a history of vaccination within 1 month, with no significant intergroup difference in the occurrence of these events. Irrespective of the presence of MOG-abs, encephalopathy including disturbance of consciousness and emotional/behavioral changes not caused by fever, systemic illness, or postictal symptoms, which is an essential condition for the diagnosis of ADEM, was present in every child. Decreased muscle strength (72.5%), fever (66.1%), and headache (50.0%) were the three most common symptoms in all the children enrolled in our study. The patients also showed seizures (24.2%), cranial nerve (III–XII) palsy (41.9%), ataxia (43.5%), optic neuritis (14.5%), sensory symptoms (32.3%), and bladder/rectum dysfunction (32.3%). By the way, the cranial nerve (III–XII) palsy described in our study was mostly considered to be of central origin. MOG-seropositive ADEM children presented with significantly lower rates of seizures (14.3 vs. 37.0%, *P* = 0.038) and cranial nerve (III–XII) palsy (25.7 vs. 63.0%, *P* = 0.003).

Among laboratory examinations, elevations of white blood cell counts were much more common in MOG-seropositive ADEM children (74.1 vs. 22.2%, *P* < 0.001), without a corresponding greater increase in other inflammatory indicators such as C-reactive protein (CRP), procalcitonin (PCT), and even ferritin and CSF leukocytes. The results of other immune-related tests, including those for autoantibodies (such as antinuclear antibodies, anti-dsDNA antibodies, and anti-neutrophil cytoplasmic antibodies), immune indicators (such as IgA, IgG, and IgM), and thyroid function tests showed no difference between the two groups. In both groups, the CSF showed features similar to viral encephalitis and aseptic encephalitis with normal or mildly elevated white blood cell counts (mainly lymphocytosis) and protein levels. Oligoclonal bands (OCBs) in CSF were only detected by a very small number of children in both groups with no difference between the groups. In addition to one child in the MOG-seropositive group, everyone else underwent tests for MOG-abs in CSF by cell-based assays. In total, 24 patients in the MOG-seropositive group also showed MOG-ab positivity in CSF and the remaining 37 children were all negative.

Moreover, although MOG-seropositive ADEM children presented with significantly lower rates of seizures, most of the patients in our study (51/62, 82.3%) underwent EEG, showing no significant difference in the abnormal rate between the two groups. Abnormal EEG findings included a slow background activity and interictal epileptic paroxysms, with no difference between the two groups. Only a very small percentage of children with seizures at onset received long-term use of antiepileptic drugs and finally developed secondary epilepsy. Visual evoked potential (VEP) was less commonly accomplished (32/62, 51.6%), also showing no significant difference.

### 3.3. MRI findings

**Figure 2:**
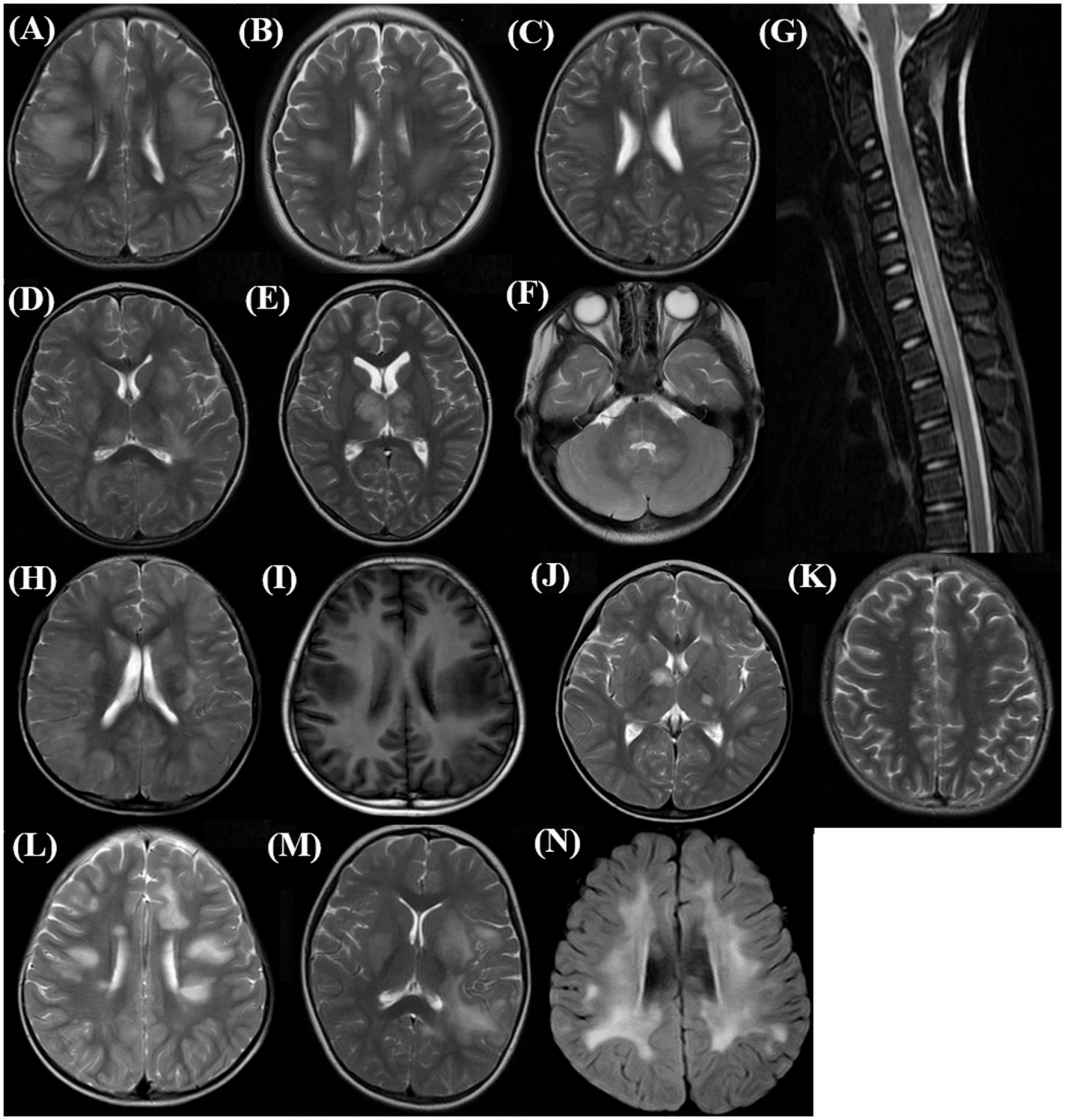
Typical and atypical imaging features in the acute phase of children with ADEM. **(A–C)** Supratentorial: large, diffuse, poorly demarcated patchy lesions mainly in subcortical white matter (Axial, T2 sequence); **(D, E)** Deep gray matter lesions (Axial, T2 sequence); **(F)** Infratentorial: brainstem and cerebellar lesions (Axial, T2 sequence); **(G)** Spinal cord long-segment lesions involving cervical and upper thoracic (Sagittal, T2 sequence); **(H)** Extensive cortical lesions (Axial, T2 sequence); **(I)** T1 hypointense lesions in supratentorial white matter (Axial, T1 sequence); **(J)** Well-demarcated lesions in deep gray matter (Axial, T2 sequence); **(K)** Small supratentorial white matter lesions (Axial, T2 sequence); **(L)** Deep white matter lesions perpendicular to the ventricle, Dawson finger type lesions (Axial, T2 sequence); **(M)** Deep periventricular white matter lesions (Axial, T2 sequence); **(N)** Large symmetric supratentorial white matter lesions (Axial, T2 sequence).

MRI is an important tool for the diagnosis of ADEM. The MRI features are summarized in Table 2. Although all patients underwent the cranial MRI to reach the diagnosis, only 56 children (56/62, 90.3%) had MRI images from our hospital, and half of them (28/56, 50.0%) also underwent spinal cord MRI. The two groups did not differ in the distributions or extent of MRI lesions. All 11 children (five in the MOG-seropositive group and six in the MOG-seronegative group) with transverse myelitis (TM) syndromes underwent spinal cord MRI. In addition, 13 children in the MOG-seropositive group and four in the MOG-seronegative group also underwent spinal cord MRI in our hospital. Although the difference was not significant (*P* = 0.209), the spinal MRI lesions in the MOG-seronegative group were more likely to present corresponding TM syndromes (6/6, 100%) than the MOG-seropositive group (5/8, 62.5%), indicating that the spinal cord lesions in MOG-seropositive were more likely to be dormant. Moreover, the MOG-seropositive group was not more likely to present with typical imaging features of ADEM (*P* = 0.090) such as large, diffusely asymmetrically distributed, poorly demarcated white matter-dominated patchy lesions and extensive involvement of deep gray matter and infratentorial areas and some parts of the spinal cord, which often presented as longitudinal extensive transverse myelitis (LETM) (Figures 2A–G). The presence and frequency of atypical features, including small, T1 hypointense, well-defined white matter lesions, cortical lesions, lesions with large symmetric white matter distribution, and paraventricular and deep white matter lesions such as Dawson's finger also did not differ significantly between the two groups (Figures 2H–N).

**Table 2:** MRI features in 56 ADEM children with and without MOG-abs from the acute phase of ADEM.

	**MOG-ab-positive (*n* = 33)**	**MOG-ab-negative (*n* = 23)**	**Z/χ^2^**	***P***
**MRI lesion distribution**, ***n*** **(%)**				
Supratentorial white matter	31 (93.9)	19 (82.6)	0.827	0.363^#^
Deep gray matter	26 (78.8)	17 (73.9)	0.181	0.671^#^
Brainstem	17 (51.5)	17 (73.9)	2.851	0.091^#^
Cerebellum	14 (42.4)	11 (47.8)	0.160	0.689^#^
Spinal (LETM)	8/18 (44.4)	6/10 (60.0)	–	0.695^§^
**Widespread score, median (IQR)**	**3 (2–4)**	**3 (2–4)**	−**0.412**	**0.680** ^*^
**Enhancement**, ***n*** **(%)**	**10/24 (41.7)**	**5/13 (38.5)**	**–**	**1.000** ^§^
**Typical brain MRI**, ***n*** **(%)**	**30 (90.0)**	**16 (69.6)**	**2.880**	**0.090** ^#^
**Atypical MRI features**, ***n*** **(%)**				
Cortical lesions	9 (27.3)	6 (26.1)	0.010	0.921^#^
Diffuse WM involvement	4 (12.7)	4 (14.1)	0.028	0.868^#^
Small lesions (< 5 mm)	1 (3.0)	4 (17.4)	1.898	0.168^#^
T1-hypointense lesions	8 (24.2)	7 (30.4)	0.265	0.607^#^
Well-defined borders	0 (0)	3 (13.0)	2.339	0.126^#^
Periventricular	12 (36.4)	10 (43.5)	0.288	0.592^#^
Dawson finger type lesions	0 (0)	1 (4.3)	–	0.411^§^
With ≤ 1 atypical MRI feature	26 (78.8)	16 (69.6)	0.615	0.433^#^

ADEM, acute disseminating encephalomyelitis; MOG-abs, myelin oligodendrocytes glycoprotein antibodies; IQR, interquartile range; MRI, magnetic resonance imaging; LETM, longitudinal extensive transverse myelitis; WM, white matter.

^§^Using Fisher's exact test.

^#^Using chi-square test.

^*^Using Mann–Whitney U-test.

### 3.4. Treatments and prognosis

All 62 children received immunotherapy based on consistent therapeutic principles in the acute phase. Of these, two patients without MOG-abs were lost to follow-up after discharge. The remaining 60 children all received at least 8 months of follow-up through outpatient and/or telephone assessments, and their features are presented in Table 3. The acute treatment options were the same regardless of the presence or absence of MOG-abs. The vast majority of children were treated with high-dose intravenous corticosteroids and intravenous immunoglobulin (IVIG), and each child received at least one of the two immunotherapies. A total of 30 children in the MOG-seropositive group and 22 in the MOG-seronegative group received both immunotherapies, while three children in each group had only intravenous corticosteroids and two in each group had IVIG only. They were supposed to take oral prednisolone and taper to stop after intravenous corticosteroids. In our study, the MOG-seropositive group received oral prednisolone tapers for a median time of 24 weeks (IQR, 11–45 weeks), which was significantly longer than the 13.5 weeks (IQR, 9.75–22.5 weeks) in the MOG-seronegative group (*P* = 0.028). Moreover, at the three-month (*P* = 0.040), six-month (*P* = 0.002), and the latest follow-up (*P* = 0.020), MOG-seropositive children showed higher levels of oral prednisone dosage with a consistent initial dose level (*P* = 0.788, 1–2 mg/kg·d). Only 12 MOG-seropositive children received long-term maintenance therapy (*P* = 0.001), which included the use of the mycophenolate mofetil (MMF, 25%), maintenance of low-dose oral prednisolone (daily dose < 0.5 mg/kg and duration > 1 year, 100%), and routine IVIG (dose of 1 g/kg and once a month, 8.3%). All 12 children received maintenance therapy with low-dose oral prednisolone, and three of them also took MMF orally. Among them, one patient changed from oral MMF to routine IVIG due to the side effects of MMF.

**Table 3:** Treatments in ADEM children with and without MOG-abs.

	**MOG-ab-positive (*n* = 35)**	**MOG-ab-negative (*n* = 27)**	**Z/χ^2^**	***P***
Onset to immunotherapy (d), median (IQR)	16 (11–21)	12 (8–18)	−1.556	0.120^*^
**Acute phase treatments**, ***n*** **(%)**				
Intravenous corticosteroids	33 (94.3)	25 (92.6)	–	–
IVIG	32 (91.4)	24 (88.9)	–	–
**Oral prednisone, median (IQR)**				
Tapering period (w)	24 (11–45)	14 (10–23)	−2.192	0.028^*^
Initial dose level	4 (4–4)	4 (4–4)	−0.269	0.788^*^
Dose level at 3 m	3 (0.25–3)	0.5 (0–2.25)	−2.052	0.040^*^
Dose level at 6 m	2 (0–2)	0 (0–0)	−3.129	0.002^*^
Dose level at latest follow-up	0 (0–0)	0 (0–0)	−2.323	0.020^*^
**Long-term maintenance therapy**, ***n*** **(%)**	**12/35 (31.4)**	**0/25 (0)**	**10.714**	**0.001** ^#^
Immunosuppressant	3 (25)	0 (0)	–	–
Rituximab	0	0	–	–
Azathioprine	0	0	–	–
Mycophenolate Mofetil	3	0	–	–
Low-dose oral prednisone	12 (100)	0 (0)	–	–
Routine IVIG	1 (8.3)	0 (0)	–	–

ADEM, acute disseminating encephalomyelitis; MOG-abs, myelin oligodendrocytes glycoprotein antibodies; IQR, interquartile range; IVIG, intravenous immunoglobulin.

Dose level of oral prednisone: 0: 0 mg/kg·d, 1: 0–0.25 mg/kg·d, 2: 0.25–0.5 mg/kg·d, 3: 0.5–1 mg/kg·d, 4: 1–2 mg/kg·d, 5: >2 mg/kg·d.

^*^Using Mann–Whitney U-test.

^#^Using chi-square test.

Differences in maintenance therapy were usually accompanied by differences in prognosis (Table 4). The median follow-up time by phone in the MOG-seropositive and MOG-seronegative groups was 29 months (IQR, 20–34 months) and 24 months (IQR, 15–34 months), respectively (*P* = 0.347), but for regular outpatient follow-up, it was 18 months (IQR, 7–26 months) in the positive group and 8 months (IQR, 6–12.5 months) in the negative group, with a significant difference (*P* = 0.003). Moreover, ADEM children with positive MOG-abs relapsed more frequently, and only this group showed nine relapse cases (*P* = 0.017). New MRI lesions appeared in these relapsed children. Some lesions appeared in the original site, and some appeared in the new area. Interestingly, at onset, seizures were not mentioned in the relapsed children, but two of them had seizures at the time of recurrence. Moreover, seven of them were diagnosed with multiphasic disseminated encephalomyelitis, and two of them were diagnosed with ADEM followed by recurrent optic neuritis. In most cases, long-term maintenance therapy was used in relapse cases (7/12, 58.3%). However, five other children who did not show relapse but had sustained high titers of MOG-abs were also treated with low-dose oral prednisone for maintenance therapy. The specific characteristics of the nine children that showed relapsed courses are presented in Table 5. Relapses usually occurred after prednisolone discontinuation (6/9, 66.7%), and some occurred during the tapering (3/9, 33.3%), but they all occurred when the daily prednisolone dose was < 0.5 mg/kg. Among these, five children had persistently positive MOG-abs, but the other four patients showed MOG-ab positivity after turning negative. None of the patients remained negative, and they were all positive at the time of recurrence.

**Table 4:** Prognosis in 60 ADEM children with and without MOG-abs.

	**MOG-ab-positive (*n* = 35)**	**MOG-ab-negative (*n* = 25)**	**t/Z/χ^2^**	***P***
Telephone FU time (m), median (IQR)	29 (20–34)	24 (15–34)	−0.941	0.347^*^
Regular FU time (m), median (IQR)	18 (7–26)	8 (6–13)	−2.948	0.003^*^
**Clinical recovery**, ***n*** **(%)**				
Complete recovery	26 (74.3)	20 (80.0)	−0.670	0.503^*^
Mild residuals	4 (11.4)	4 (16.0)		
Moderate/severe residuals	5 (14.3)	1 (4.0)		
Death	0 (0)	0 (0)		
**MRI recovery status, median (IQR)**				
At 3 m	3 (2–3)	2 (2–3)	−0.927	0.354^*^
At 6 m	2 (1.75–3)	2 (1–2)	−1.905	0.057^*^
At latest FU	2 (1–2.5)	1 (1–2)	−1.577	0.115^*^
**Neurological sequelae**, ***n*** **(%)**				
Secondary epilepsy	5 (14.3)	1 (4.0)	0.762	0.383^#^
Mental disorders	0 (0)	1 (4.0)	–	0.417^§^
Motor dysfunction	1 (2.9)	2 (8.0)	0.090	0.764^#^
Cognitive impairment	8 (22.9)	3 (12.0)	0.538	0.463^#^
**Relapse**, ***n*** **(%)**	**9 (25.7)**	**0 (0)**	**5.681**	**0.017** ^#^
**EDSS scores at onset**, $\bar{X}\;\pm$**SD**	**3.8** ±**1.9**	**4.9** ±**2.4**	−**2.447**	**0.017**
**mRS scores at onset, median (IQR)**	**3 (2–4)**	**4 (3–5)**	−**2.235**	**0.025** ^*^
**EDSS scores at 6 m, median (IQR)**	**1.0 (0–1.5)**	**1.0 (0–2.0)**	−**0.323**	**0.747** ^*^
**mRS scores at 6 m, median (IQR)**	**0 (0–0)**	**0 (0–1)**	−**0.640**	**0.522** ^*^
**EDSS scores at latest FU, median (IQR)**	**1.0 (0–1.5)**	**1.0 (0–1.5)**	−**0.485**	**0.628** ^*^
**mRS scores at latest FU, median (IQR)**	**0 (0–0)**	**0 (0–1)**	−**0.607**	**0.544** ^*^

ADEM, acute disseminating encephalomyelitis; MOG-abs, myelin oligodendrocytes glycoprotein antibodies; FU, follow-up; IQR, interquartile range; MRI, magnetic resonance imaging; EDSS, Expanded Disability Status Scale; mRS, modified Rankin Scale.

MRI recovery status: 1: Complete resolution; 2: Minor residuals; 3: Moderate residuals; 4: Marked residuals.

Using t-test.

^*^Using Mann–Whitney U test.

^§^Using Fisher's exact test.

^#^Using chi-square test.

**Table 5:** Partial characteristics of nine relapsed ADEM children with MOG-abs.

**No**.	**Sex**	**Year of onset (y)**	**ARR**	**Time from first recurrence to onset (m)**	**Prednisone dose at relapse (mg/kg·d)**	**MOG-ab status**	**Maintenance therapy**
1	Female	6	0.32	24	0 (Medication for 6 m)	P-N-P	Prednisone
2	Male	3	0.50	6	0 (Medication for 4 m)	All P	–
3	Female	4	0.41	7	0.25–0.5 (Medication for 6 m)	All P	Prednisone
4	Male	6	0.50	6	0.25–0.5 (Medication for 4 m)	P-N-P	Prednisone + MMF
5	Male	7	0.40	21	0 (Medication for 12 m)	All P	Prednisone + Routine IVIG/MMF
6	Female	5	0.33	35	0 (Medication for 6 m)	P-N-P	Prednisone
7	Female	11	0.67	10	0 (Medication for 4 m)	P-N-P	–
8	Female	5	0.92	6	0–0.25 (Medication for 5 m)	All P	Prednisone
9	Female	10	1.5	13	0 (Medication for 8 m)	All P	Prednisone + MMF

ADEM, acute disseminating encephalomyelitis; MOG, myelin oligodendrocytes glycoprotein; ARR, annual relapse rate; MMF, mycophenolate mofetil; IVIG, intravenous immunoglobulin; P-N-P, positive-negative-positive; All P, persistent positive; y, year; m, month.

The two groups showed no significant differences in clinical recovery at the latest follow-up and the MRI recovery status at 3-, 6-month, or the latest follow-up (median follow-up period, 12 months; IQR 6–18 months). The evaluation of neurological dysfunction status by EDSS and mRS scores showed that the MOG-seropositive group had lower EDSS and mRS scores (*P* = 0.017 and *P* = 0.025) than the negative group at onset, indicating less severe disability. However, at the 6-month or the latest follow-up, the two groups showed no difference. Our study also followed up on neurological sequelae, showing no significant difference between the two groups in the incidence of secondary epilepsy, mental disorders, motor impairment, and cognitive impairment. Cognitive impairment was the most common neurological sequelae of all ADEM children in our study (11/60, 18.3%), and it mainly manifested as a slight impact on learning described by parents during follow-up, such as poor academic performance, lack of attention concentration, and so on, and the damage was often not severe and rarely affected their daily life heavily. However, more specific assessments were not possible through the current retrospective approach.

## 4. Discussion

ADEM is a rare CNS demyelinating disease commonly seen in children, with a reported incidence of 0.1–0.3 per 1,00,000 children per year (Banwell et al., 2009; Absoud et al., 2013). The difficulties in the clinical diagnosis and treatment of ADEM are mainly attributable to the lack of relevant specific biomarkers (Koelman et al., 2016), especially biomarkers that can predict the prognosis (Menge et al., 2007). Distinguishing ADEM early from other CNS demyelinating diseases and predicting the prognosis are challenging, limiting the benefits of early interventions to improve the prognosis. Before the IPMSSG updated the specific diagnostic criteria for pediatric ADEM in 2013 (Krupp et al., 2013), overdiagnosis was mainly based on clinical features that frequently occurred in clinical and even research settings (Young et al., 2010). MOG-abs were discovered in acquired demyelinating syndromes for the first time more than a decade previously, but since 2015, MOG antibody testing has become widely available (Salama et al., 2019), and accurate detection of MOG-abs by cell-based assays was officially recommended in 2018 (Jarius et al., 2018). As a result, our understanding of MOG-abs has continued to show rapid advancements. Studies have shown a close association between MOG-abs and ADEM and its recurrent forms. MOG-abs are present in more than 50% of children with ADEM (Duignan et al., 2018) and almost all of the children with multiphasic disseminated encephalomyelitis. The MOG-ab status was proved to be associated with the course of ADEM since the expression of these antibodies usually declined or even disappeared after complete recovery from ADEM (Di Pauli et al., 2011), and a continued positive state was more likely to indicate relapse (Waters et al., 2020). However, the potential of MOG-abs to predict the prognosis in ADEM remains controversial (Duignan et al., 2018), and treatment guidance solely based on the MOG-ab status at a certain time can be difficult.

The existing studies have paid little attention to ADEM patients without MOG-abs, and there is no consensus on the differences between them and MOG-seropositive children. Moreover, the existing retrospective comparative studies were characterized by insufficient sample sizes, inconsistent conclusions, and a lack of detailed descriptions and comparisons of treatments and prognosis (Baumann et al., 2015; Zhang et al., 2020; Lei et al., 2022; Shen et al., 2022). As a retrospective study with a relatively large sample size, our study provided information on the differences between MOG-seropositive and MOG-seronegative children in terms of clinical and imaging characteristics. It is also the first study to highlight differences in treatments, follow-up, and prognosis.

Comparison of the clinical features of MOG-seropositive and MOG-seronegative children yielded varied conclusions in different studies. Zhang et al. (2020) reported that MOG-seropositive children with ADEM tended to show more ataxia (*P* = 0.025), while MOG-seronegative children tended to show higher rates of movement disorders such as paralysis (*P* = 0.004) and rectal/bladder dysfunction (*P* = 0.035). Shen et al. (2022) showed that the seropositive group had significantly more symptoms of meningeal involvement (*P* = 0.008). Nevertheless, two other studies showed no differences in the range of clinical symptoms between ADEM children with and without MOG-abs (Baumann et al., 2015; Lei et al., 2022). Among them, Lei et al. (2022) proposed that children in the seropositive group had a significantly lower proportion of prodromal infections than those in the negative group (*P* = 0.017). However, in our study, the MOG-seropositive ADEM children were found to present fewer seizures and lower rates of cranial nerve (III–XII) palsy than seronegative children. Generally, the differences in clinical manifestations were non-specific.

MOG-abs are known to play an important role in the autoimmune pathogenesis of ADEM (Fernandez-Carbonell et al., 2016; Chen et al., 2018). Our study showed no significant intergroup difference in the detection of routine immune indicators. MOG-seropositive ADEM children showed more elevated white blood cell counts, which may indicate more inflammatory responses in the body. However, inflammatory indicators such as procalcitonin and CRP levels did not show simultaneous relative increments, which may be a point of differentiation from bacterial infection. Furthermore, CSF examinations showed no significant differences, with both groups showing a tendency toward a slight increase in white blood cell counts, mainly lymphocytes, that was sometimes accompanied by a slight increase in protein levels and was easily confused with viral encephalitis and aseptic encephalitis.

Typical cranial MRI features are also one of the essential criteria for the diagnosis of ADEM (Krupp et al., 2013). In our study, the two groups showed no significant differences in terms of lesion distributions and lesion extent on cranial MRI or the appearance of typical/atypical lesions. However, in a study conducted in 2015 (Baumann et al., 2015), the distribution range of MRI lesions in MOG-seropositive ADEM was wider (*P* = 0.035), and ADEM children without MOG-abs were more likely to have well-defined atypical lesions (*P* = 0.008) and showed more atypical MRI features (*P* = 0.003). Another study showed that the MOG-seropositive children with ADEM had more lesions in the atypical area of the cortex (*P* = 0.012) (Lei et al., 2022). But consistent with our study, Zhang et al. (2020) reported similar frequencies of typical MRI features in the two groups without analysis of atypical features. In addition, no other studies have compared typical or atypical MRI features between MOG-seropositive and MOG-seronegative ADEM children. With regard to MRI lesion distributions, Zhang et al. (2020) reported greater involvement of the cerebellum (*P* = 0.045) in MOG-seropositive ADEM children, probably explaining why the children with MOG-abs in their study more frequently presented with ataxia clinically. In addition, for MOG-seropositive ADEM, Lei et al. (2022) reported more lesions in the thalamus (*P* = 0.022) and Shen et al. (2022) reported more lesions in the frontal lobe (*P* = 0.019) although both studies did not compare the breadth of lesion distributions. Some of the abovementioned variable conclusions explain the differences in clinical characteristics, but most of them were non-specific and made little sense for clinical practice. Moreover, with the diagnostic criteria of ADEM gradually becoming stricter, patients with many atypical MRI lesions are not diagnosed as ADEM.

Although children without clinical symptoms of TM syndromes rarely underwent spinal cord MRI clinically, we managed to perform spinal cord MRI in half of our pediatric patients with ADEM, including 17 patients without TM symptoms. Although the two groups showed no significant difference in the proportion of abnormal spinal MRI scans, the spinal lesions obtained from MOG-seropositive ADEM children were more likely to be subclinical and tended to present fewer TM symptoms. This tendency was similarly confirmed in previous studies. One study reported that spinal cord MRI involvement was more common in MOG-seropositive ADEM children (*P* = 0.003), with a smaller proportion of spinal cord MRI lesions accompanied by corresponding TM symptoms (61.5 vs. 100%), and the two groups showed no difference in the appearance of both TM symptoms and MRI lesions (*P* = 0.286) (Baumann et al., 2015). Another study revealed the same tendency (28.6 vs. 100%) with a similar frequency of spinal cord MRI lesions (*P* = 0.660) but significantly more lesions with TM symptoms in MOG-seronegative children (*P* = 0.035) (Zhang et al., 2020). Although data from more studies are required to confirm this phenomenon, for now, considering the findings showing spinal cord lesions in ~1/3 of ADEM patients (Pohl et al., 2016), spinal cord MRI should also receive attention and should be more frequently recommended in clinical practice. We recommend that spinal cord MRI is essential for ADEM children, especially those with MOG-abs, even in the absence of TM symptoms.

Although seizures were more common in children without MOG-abs in our study, epileptic discharges were not more evident in EEG, indicating that most of the clinical seizures in ADEM children were not accompanied by abnormal EEG discharges. Similarly, in a recent retrospective study, symptomatic seizures were reported to show a self-limiting course in MOGAD and MOG-seropositive ADEM, and most of the patients showed seizure freedom at the last follow-up (Montalvo et al., 2022). This finding may explain why seizures are one of the most common clinical symptoms of ADEM, but only a small proportion of children eventually develop secondary epilepsy or post-ADEM epilepsy and require maintenance therapy with antiepileptic drugs (Rossor et al., 2020).

Immunotherapy is the generally agreed principle of acute phase treatments for ADEM. Most of the children in our study received intravenous corticosteroid therapy and IVIG. The difference started from oral prednisolone tapering following the use of intravenous corticosteroids. In addition to requiring a consistent initial dose level of oral prednisolone, MOG-seropositive ADEM children also required slower oral prednisolone tapering than the negative group, which was reflected by the duration of steroid cessation and the dose levels at the third month, the sixth month, and the latest follow-up. Only 12 children in the MOG-seropositive group accepted long-term maintenance therapy. We hold the opinion that these differences in treatments are probably attributable to the different MOG-ab statuses and follow-up findings for recurrence and prognosis.

The two groups showed no differences in telephone follow-up durations, but the regular outpatient follow-up duration for MOG-seropositive children was significantly longer because even if the clinical symptoms and MRI lesions were greatly relieved, MOG-seropositive children still underwent regular follow-up often until MOG-abs turned negative, while the MOG-seronegative children rarely visited the outpatient clinic for follow-up after the symptoms were relieved or underwent retesting for MOG-abs unless they recovered poorly or showed fluctuating signs and symptoms. During at least 8 months of follow-up after onset, the recurrence rate in the MOG-seropositive group was significantly higher than that in the MOG-seronegative group, in agreement with the results of a previous study (*P* = 0.030) (Shen et al., 2022). During the follow-up evaluations of children showing relapse, we found that all relapses occurred when the daily dose of oral prednisolone was < 0.5 mg/kg or even after withdrawal, which was consistent with a previous report on overall MOGAD (Reindl and Waters, 2019). Moreover, patients with immunotherapy lasting < 3 months were twice as likely to relapse as those treated for a longer period (Jurynczyk et al., 2017; Hacohen et al., 2018; Ramanathan et al., 2018), explaining why MOG-seropositive children should undergo longer and careful oral prednisolone tapering. In addition, five children showing persistently positive MOG-abs required long-term oral prednisolone maintenance therapy despite showing no clinical recurrence in consideration of the higher risk of recurrence in children with persistently positive MOG-abs (López-Chiriboga et al., 2018; Waters et al., 2020). After a period of stable follow-up without recurrence, even if the MOG-abs continued to be positive, we usually considered a slow withdrawal of oral prednisolone in clinical practice, with the total course of steroid use ranging from 15 to 33 months.

Most of the relapsed children received long-term maintenance therapy. In addition to low-dose oral prednisolone maintenance, three of them took immunosuppressive drugs or accepted routine IVIG at the same time. It remains unclear whether the presence of MOG-abs is a signal for the initiation of long-term immunosuppressive therapy (Reindl et al., 2013). Some believe that patients with MOG-ab seropositivity do not require immediate initiation of immunosuppressive therapy (Hacohen et al., 2015; Waters et al., 2020). Although our study and a previous study both showed that MOG-seropositive ADEM children were easier to relapse than those without MOG-abs (Shen et al., 2022), only a very small percentage of them showed repeated relapses or serious sequelae. Usually, temporary follow-up and observation were preferred instead of immediate immunosuppressive therapy for patients who relapsed for the first time (Hino-Fukuyo et al., 2019). Only 1/3 of the recurrent children in our study required immunosuppressive therapy, while the others received steroid therapy alone. In contrast, another study proposed that the use of long-term immunosuppression was required once clinical relapse occurred in any MOGAD (Jarius et al., 2016). In ADEM children who tested persistently positive for serum MOG-abs, immunosuppressive therapy was recommended to prevent recurrence (López-Chiriboga et al., 2018). However, from our study, although persistently positive MOG-abs are a reason for long-term low-dose oral prednisolone, the clinical use of immunosuppressants is currently only considered in relapsed children.

No significant difference was observed between MOG-seropositive and MOG-seronegative ADEM children either in clinical recovery at the latest follow-up and MRI recovery at the third month, the sixth month, or the latest follow-up. The EDSS and mRS assessments of the neurological dysfunction status at onset and during follow-up showed that the disability of the MOG-seropositive ADEM children at onset was less severe than that of MOG-seronegative children, with no difference at the sixth month or the last follow-up. Thus, despite the differences in disability status at onset, the children in the two groups showed similar recovery outcomes in agreement with a previous study that performed evaluations by the mRS score alone (Zhang et al., 2020). Another study published a few years ago indicated that MOG-seropositive ADEM children had a better eventual clinical recovery than MOG-seronegative children (*P* = 0.038), without comparing the status at onset (Baumann et al., 2015). The persistence of MOG-abs was proved to be a sign of relapse, incomplete recovery, and steroid dependence in acquired demyelinating syndromes (Di Pauli et al., 2011), but some of the patients also showed a chance of no recurrence and satisfactory recovery. Moreover, due to the insufficient analysis of the consecutive and dynamic changes of MOG-ab status and titers, it was difficult to simply conclude that MOG-seropositive children with ADEM had a slower recovery than seronegative children in our study since the MOG-ab titer data obtained during the follow-up were not enough for analysis in this retrospective study.

The two groups showed no significant differences in the incidence of neurological sequelae: secondary epilepsy, mental illness, movement disorders, and cognitive impairment. Cognitive impairment was the most common sequelae in ADEM children in this study. Approximately 33% of children with ADEM showed cognitive impairment or behavioral changes at follow-up in a previous study (Ketelslegers et al., 2011). Unfortunately, there is no unified scale or questionnaire for more detailed and accurate assessments of the occurrence and severity of cognitive impairment in children with ADEM at present, and most of the information in our study was obtained from oral interactions with the children's parents.

Our study demonstrated the differences in clinical manifestations, radiological features, treatments, and prognosis between MOG-seropositive and MOG-seronegative ADEM children, and we described the differences in follow-up and prognosis in detail for the first time. The limitation of our study is that as a retrospective study, some of the auxiliary examination data were missing or unavailable, and some data were obtained through telephone follow-up, which could have easily led to some bias, and the dynamic evaluation of MOG-ab titers was not completed. Therefore, our conclusions can provide a reference for clinical practice and further research but are insufficient to guide clinical decision-making at present. Existing reports focusing on the differences in clinical and MRI characteristics have provided some controversial conclusions as a result of different subjects and sample sizes and are limited by the retrospective approach of this study. In the future, more cross-sectional and prospective studies using large samples from multiple centers as well as detailed and scientific prospective follow-up assessments for the prognosis are required to draw conclusions of a higher evidence-based level and resolve these disputes. We look forward to the development of more optimized treatment plans and more scientific and formal follow-ups in the future.

## 5. Conclusion

In conclusion, in comparison with the MOG-seronegative ADEM children, MOG-seropositive ADEM children showed lower rates of seizures and cranial nerve (III-XII) palsy with more obvious isolated white blood cell elevations in the blood. Since the two groups showed no significant difference in other typical clinical features, early identification of the groups was difficult. Spinal MRI lesions in MOG-seropositive ADEM children seemed to show subclinical characteristics. For these patients, we recommended routine spinal cord MRI examinations. Moreover, children with positive MOG-abs were more likely to show relapse and showed a longer period of oral prednisone tapering with greater use of immunosuppressive agents. While MOG-seronegative ADEM children had more severe neurological impairment at onset, they showed no significant difference after recovery.

## Data availability statement

The raw data supporting the conclusions of this article will be made available by the authors, without undue reservation.

## Ethics statement

The studies involving human participants were reviewed and approved by the Ethics Committee of Children's Hospital of Chongqing Medical University. Written informed consent to participate in this study was provided by the participants' legal guardian/next of kin. Written informed consent was obtained from the individual(s), and minor(s)' legal guardian/next of kin, for the publication of any potentially identifiable images or data included in this article.

## Author contributions

XD and XL contributed to the conception and design of the study with suggestions from LJ, YJ, and PY. YJ and PY helped to develop a specific research schedule. XD, PW, and JM collected and analyzed the data. XF guided the analysis of MRI images. XD drafted the initial manuscript, which was edited by LJ and XL. All authors approved the final version of the manuscript.

## References

[B1] Absoud M. Lim M. J. Chong W. K. De Goede C. G. Foster K. Gunny R.. (2013). Paediatric acquired demyelinating syndromes: incidence, clinical and magnetic resonance imaging features. Mult. Scler. 19, 76–86. 10.1177/1352458512445944 22516794 PMC3409874

[B2] Banwell B. Kennedy J. Sadovnick D. Arnold D. L. Magalhaes S. Wambera K.. (2009). Incidence of acquired demyelination of the CNS in Canadian children. Neurology 72, 232–239. 10.1212/01.wnl.0000339482.84392.bd 19153370

[B3] Baumann M. Sahin K. Lechner C. Hennes E. M. Schanda K. Mader S.. (2015). Clinical and neuroradiological differences of paediatric acute disseminating encephalomyelitis with and without antibodies to the myelin oligodendrocyte glycoprotein. J. Neurol. Neurosurg. Psychiatry 86, 265–272. 10.1136/jnnp-2014-308346 25121570

[B4] Brenton J. N. Banwell B. L. (2016). Therapeutic approach to the management of pediatric demyelinating disease: multiple sclerosis and acute disseminated encephalomyelitis. Neurotherapeutics 13, 84–95. 10.1007/s13311-015-0396-0 26496907 PMC4720662

[B5] Bruijstens A. L. Lechner C. Flet-Berliac L. Deiva K. Neuteboom R. F. Hemingway C.. (2020). E.U. paediatric MOG consortium consensus: part 1 - classification of clinical phenotypes of paediatric myelin oligodendrocyte glycoprotein antibody-associated disorders. Eur. J. Paediatr. Neurol. 29, 2–13. 10.1016/j.ejpn.2020.10.006 33162302

[B6] Chen Y. Ma F. Xu Y. Chu X. Zhang J. (2018). Vaccines and the risk of acute disseminated encephalomyelitis. Vaccine 36, 3733–3739. 10.1016/j.vaccine.2018.05.063 29784468

[B7] Cole J. Evans E. Mwangi M. Mar S. (2019). Acute disseminated encephalomyelitis in children: an updated review based on current diagnostic criteria. Pediatr. Neurol. 100, 26–34. 10.1016/j.pediatrneurol.2019.06.017 31371120

[B8] Di Pauli F. Mader S. Rostasy K. Schanda K. Bajer-Kornek B. Ehling R.. (2011). Temporal dynamics of anti-MOG antibodies in CNS demyelinating diseases. Clin. Immunol. 138, 247–254. 10.1016/j.clim.2010.11.013 21169067

[B9] Duignan S. Wright S. Rossor T. Cazabon J. Gilmour K. Ciccarelli O.. (2018). Myelin oligodendrocyte glycoprotein and aquaporin-4 antibodies are highly specific in children with acquired demyelinating syndromes. Dev. Med. Child Neuril. 60, 958–962. 10.1111/dmcn.13703 29468668

[B10] Fernandez-Carbonell C. Vargas-Lowy D. Musallam A. Healy B. McLaughlin K. Wucherpfennig K. W.. (2016). Clinical and MRI phenotype of children with MOG antibodies. Mult. Scler. 22, 174–184. 10.1177/1352458515587751 26041801 PMC4669239

[B11] Hacohen Y. Absoud M. Deiva K. Hemingway C. Nytrova P. Woodhall M.. (2015). Myelin oligodendrocyte glycoprotein antibodies are associated with a non-MS course in children. Neurol. Neuroimmunol. Neuroinflamm. 2, e81. 10.1212/NXI.0000000000000081 25798445 PMC4360800

[B12] Hacohen Y. Wong Y. Y. Lechner C. Jurynczyk M. Wright S. Konuskan B.. (2018). Disease course and treatment responses in children with relapsing myelin oligodendrocyte glycoprotein antibody-associated disease. JAMA Neurol. 75, 478–487. 10.1001/jamaneurol.2017.4601 29305608 PMC5885190

[B13] Hennes E. M. Baumann M. Schanda K. Anlar B. Bajer-Kornek B. Blaschek A.. (2017). Prognostic relevance of MOG antibodies in children with an acquired demyelinating syndrome. Neurology 89, 900–908. 10.1212/WNL.0000000000004312 28768844

[B14] Hino-Fukuyo N. Haginoya K. Takahashi T. Nakashima I. Fujihara K. Takai Y.. (2019). Long-term outcome of a group of Japanese children with myelin-oligodendrocyte glycoprotein encephalomyelitis without preventive immunosuppressive therapy. Brain Dev. 41, 790–795. 10.1016/j.braindev.2019.06.004 31281008

[B15] Jarius S. Paul F. Aktas O. Asgari N. Dale R. C. de Seze J.. (2018). MOG encephalomyelitis: international recommendations on diagnosis and antibody testing. J. Neuroinflamm. 15, 134. 10.1186/s12974-018-1144-2 29724224 PMC5932838

[B16] Jarius S. Ruprecht K. Kleiter I. Borisow N. Asgari N. Pitarokoili K.. (2016). MOG-IgG in NMO and related disorders: a multicenter study of 50 patients. Part 2: epidemiology, clinical presentation, radiological and laboratory features, treatment responses, and long-term outcome. J. Neuroinflamm. 13, 280. 10.1186/s12974-016-0718-0 27793206 PMC5086042

[B17] Jurynczyk M. Messina S. Woodhall M. R. Raza N. Everett R. Roca-Fernandez A.. (2017). Clinical presentation and prognosis in MOG-antibody disease: a UK study. Brain 140, 3128–3138. 10.1093/brain/awx276 29136091

[B18] Ketelslegers I. A. Visser I. E. Neuteboom R. F. Boon M. Catsman-Berrevoets C. E. Hintzen R. Q.. (2011). Disease course and outcome of acute disseminated encephalomyelitis is more severe in adults than in children. Mult. Scler. 17, 441–448. 10.1177/1352458510390068 21148017

[B19] Koelman D. L. Chahin S. Mar S. S. Venkatesan A. Hoganson G. M. Yeshokumar A. K.. (2016). Acute disseminated encephalomyelitis in 228 patients: a retrospective, multicenter US study. Neurology 86, 2085–2093. 10.1212/WNL.0000000000002723 27164698

[B20] Krupp L. B. Tardieu M. Amato M. P. Banwell B. Chitnis T. Dale R. C.. (2013). International Pediatric Multiple Sclerosis Study Group criteria for pediatric multiple sclerosis and immune-mediated central nervous system demyelinating disorders: revisions to the 2007 definitions. Mult. Scler. 19, 1261–1267. 10.1177/1352458513484547 23572237

[B21] Leake J. A. Albani S. Kao A. S. Senac M. O. Billman G. F. Nespeca M. P.. (2004). Acute disseminated encephalomyelitis in childhood: epidemiologic, clinical and laboratory features. Pediatr. Infect. Dis. J. 23, 756–764. 10.1097/01.inf.0000133048.75452.dd 15295226

[B22] Lei M. Cui Y. Dong Z. Zhi X. Shu J. Cai C.. (2022). Clinical and magnetic resonance imaging characteristics of pediatric acute disseminating encephalomyelitis with and without antibodies to myelin oligodendrocyte glycoprotein. Front Pediatr. 10, 859932. 10.3389/fped.2022.859932 35669399 PMC9163708

[B23] López-Chiriboga A. S. Majed M. Fryer J. Dubey D. McKeon A. Flanagan E. P.. (2018). Association of MOG-IgG serostatus with relapse after acute disseminated encephalomyelitis and proposed diagnostic criteria for MOG-IgG-associated disorders. JAMA Neurol. 75, 1355–1363. 10.1001/jamaneurol.2018.1814 30014148 PMC6248120

[B24] Menge T. Hemmer B. Nessler S. Wiendl H. Neuhaus O. Hartung H. P.. (2005). Acute disseminated encephalomyelitis: an update. Arch. Neurol. 62, 1673–1680. 10.1001/archneur.62.11.1673 16286539

[B25] Menge T. Kieseier B. C. Nessler S. Hemmer B. Hartung H. P. Stüve O.. (2007). Acute disseminated encephalomyelitis: an acute hit against the brain. Curr. Opin. Neurol. 20, 247–254. 10.1097/WCO.0b013e3280f31b45 17495616

[B26] Mikaeloff Y. Adamsbaum C. Husson B. Vallée L. Ponsot G. Confavreux C.. (2004). MRI prognostic factors for relapse after acute CNS inflammatory demyelination in childhood. Brain 127, 1942–1947. 10.1093/brain/awh218 15289266

[B27] Mikaeloff Y. Caridade G. Husson B. Suissa S. Tardieu M. (2007). Acute disseminated encephalomyelitis cohort study: prognostic factors for relapse. Eur. J. Paediatr. Neurol. 11, 90–95. 10.1016/j.ejpn.2006.11.007 17188007

[B28] Montalvo M. Khattak J. F. Redenbaugh V. Britton J. Sanchez C. V. Datta A.. (2022). Acute symptomatic seizures secondary to myelin oligodendrocyte glycoprotein antibody-associated disease. Epilepsia. 63, 3180–3191. 10.1111/epi.17424 36168809 PMC10641900

[B29] Otallah S. (2020). Acute disseminated encephalomyelitis in children and adults: a focused review emphasizing new developments. Mult. Scler. 27, 1153–1160. 10.1177/1352458520929627 32552256

[B30] Pohl D. Alper G. Van Haren K. Kornberg A. J. Lucchinetti C. F. Tenembaum S.. (2016). Acute disseminated encephalomyelitis: updates on an inflammatory CNS syndrome. Neurology 87, S38–45. 10.1212/WNL.0000000000002825 27572859

[B31] Ramanathan S. Mohammad S. Tantsis E. Nguyen T. K. Merheb V. Fung V. S. C.. (2018). Clinical course, therapeutic responses and outcomes in relapsing MOG antibody-associated demyelination. J. Neurol. Neurosurg. Psychiatry 89, 127–137. 10.1136/jnnp-2017-316880 29142145 PMC5800335

[B32] Reindl M. Di Pauli F. Rostásy K. Berger T. (2013). The spectrum of MOG autoantibody-associated demyelinating diseases. Nat. Rev. Neurol. 9, 455–461. 10.1038/nrneurol.2013.118 23797245

[B33] Reindl M. Waters P. (2019). Myelin oligodendrocyte glycoprotein antibodies in neurological disease. Nat. Rev. Neurol. 15, 89–102. 10.1038/s41582-018-0112-x 30559466

[B34] Rossor T. Benetou C. Wright S. Duignan S. Lascelles K. Robinson R.. (2020). Early predictors of epilepsy and subsequent relapse in children with acute disseminated encephalomyelitis. Mult. Scler. 26, 333–342. 10.1177/1352458518823486 30730236

[B35] Salama S. Khan M. Pardo S. Izbudak I. Levy M. M. O. G. (2019). antibody-associated encephalomyelitis/encephalitis. Mult. Scler. 25, 1427–1433. 10.1177/1352458519837705 30907249 PMC6751007

[B36] Shen J. Lin D. Jiang T. Gao F. Jiang K. (2022). Clinical characteristics and associated factors of pediatric acute disseminated encephalomyelitis patients with MOG antibodies: a retrospective study in Hangzhou, China. BMC Neurol. 22, 418. 10.1186/s12883-022-02963-0 36352355 PMC9644585

[B37] Waters P. Fadda G. Woodhall M. O'Mahony J. Brown R. A. Castro D. A.. (2020). Serial anti-myelin oligodendrocyte glycoprotein antibody analyses and outcomes in children with demyelinating syndromes. JAMA Neurol. 77, 82–93. 10.1001/jamaneurol.2019.2940 31545352 PMC6763982

[B38] Wong Y. Y. M. Hacohen Y. Armangue T. Wassmer E. Verhelst H. Hemingway C.. (2018). Paediatric acute disseminated encephalomyelitis followed by optic neuritis: disease course, treatment response and outcome. Eur. J. Neurol. 25, 782–786. 10.1111/ene.13602 29443442

[B39] Young N. P. Weinshenker B. G. Parisi J. E. Scheithauer B. Giannini C. Roemer S. F.. (2010). Perivenous demyelination: association with clinically defined acute disseminated encephalomyelitis and comparison with pathologically confirmed multiple sclerosis. Brain 133, 333–348. 10.1093/brain/awp321 20129932 PMC2822631

[B40] Zhang M. Shen J. Zhou S. Du X. Li W. Yu L.. (2020). Clinical and Neuroimaging characteristics of pediatric acute disseminating encephalomyelitis with and without antibodies to myelin oligodendrocyte glycoprotein. Front. Neurol. 11, 593287. 10.3389/fneur.2020.593287 33329345 PMC7717994

